# Low-frequency monitoring for community clozapine initiations: A comparative study relative to standard frequency assessments

**DOI:** 10.1177/02698811231171532

**Published:** 2023-05-12

**Authors:** Yuya Mizuno, Devi L. Bridglal, Jack Coumbe, Hari McGrath, Ayush Adhikari, Emma Butler, Ilaria Bonoldi, David Taylor, Oliver D. Howes

**Affiliations:** 1 Department of Psychosis Studies, Institute of Psychiatry, Psychology & Neuroscience, King’s College London, London, UK; 2 TREAT Service, South London and Maudsley NHS Foundation Trust, London, UK; 3 Department of Neuropsychiatry, Keio University School of Medicine, Tokyo, Japan; 4 GKT School of Medical Education, King’s College London, London, UK; 5 Department of Pharmacy, Maudsley Hospital, South London and Maudsley NHS Foundation Trust, London, UK; 6 Institute of Pharmaceutical Science, King’s College London, London, UK; 7 Psychiatric Imaging Group, Medical Research Council London Institute of Medical Sciences, Hammersmith Hospital, London, UK; 8 Institute of Clinical Sciences, Faculty of Medicine, Imperial College London, UK

Dear Editor,

Clozapine is currently the only licensed medication for treatment-resistant schizophrenia (Kane et al., 2019). In the UK, the importance of early access to clozapine treatment has been recognised nationally (National Institute for Health and Care Excellence, 2016). Benefits of initiating clozapine in the community include reductions in symptom severity, frequency of healthcare use, and medical costs (Butler et al., 2022). However, during initiation of clozapine, there are risks of side effects including orthostatic hypotension, sedation, constipation, hypersalivation, symptomatic tachycardia, and fever, as well as more rare but serious adverse effects such as myocarditis and agranulocytosis (Correll et al., 2022; Taylor et al., 2021). Consequently, treatment guidelines recommend frequent monitoring of postural blood pressure, heart rate, temperature, and respiratory rate during the initial 4 weeks of clozapine initiation (Correll et al., 2022; Taylor et al., 2021). This monitoring is considered essential to detect and manage potential side effects, and to reduce early discontinuation of treatment (Correll et al., 2022; Taylor et al., 2021).

During the COVID-19 pandemic, the risk of patients and staff being exposed to, or transmitting SARS-CoV-2 required face-to-face contact to be minimised where feasible and appropriate. In this context, an international consensus statement recommended reduced frequency of blood monitoring for patients stable on clozapine treatment for over 1 year (Siskind et al., 2020). However, strategies to reduce face-to-face contact in patients initiating clozapine during the COVID-19 pandemic have not been reported. To maintain access to clozapine treatment whilst minimising face-to-face contact, we developed a low-frequency physical monitoring protocol which reduced in-person contact by approximately 40% during the first 3 weeks of community clozapine initiations (see Supplemental Table 1 for overview). Current guidelines recommend in-person reviews 5 days/week for physical observations during Weeks 1–2, followed by 3 days/week for Week 3 (Beck et al., 2014; Taylor et al., 2021). In contrast, our protocol reduced in-person reviews to 3 days/week during Weeks 1–2, and 2 days/week for Week 3. The frequency of physical monitoring did not differ between protocols after Week 3.

To evaluate the safety of this low-frequency physical monitoring (LFM) protocol, we carried out a retrospective cohort study comparing clinical outcomes to patients receiving standard-frequency monitoring (SFM) before the pandemic. To test if LFM resulted in poorer detection and management of side effects and ultimately treatment failure, we chose all-cause discontinuation of clozapine as our primary outcome. As secondary outcomes, we compared the incidence of hospitalisation due to clozapine-related side effects, and the incidence of side effects requiring physical monitoring for detection (i.e. hypotension, hypertension, tachycardia, and fever). We hypothesised that our LFM protocol would be as safe and effective as SFM in preventing all-cause discontinuation of clozapine.

The study was part of a clinical audit of patients undergoing community clozapine initiations with the Treatment Review and Assessment Team (TREAT) service (Beck et al., 2014). We reviewed the medical notes of all 16 patients who initiated clozapine under the LFM protocol between March 2020 to August 2021. A control group was selected from a pool of 70 patients who initiated clozapine under our SFM protocol between June 2011 to November 2019. Controls (*n* = 16) were selected blind to outcome and individually matched to cases based on sex, age (±5 years), and smoking status, because these factors may influence clozapine titration by affecting its metabolism (Rostami-Hodjegan et al., 2004). In-person reviews by clinicians involved face-to-face assessments including physical observations (sitting and standing blood pressure, heart rate, SpO2, temperature, and respiratory rate), side-effect and mental state review. The assessment conducted during the in-person reviews were identical between the LFM and SFM protocols (i.e. they only differed in frequency during the first 3-weeks of treatment). For patients who used tobacco, we encouraged them to report any changes in smoking habits during our in-person reviews due to its known impact on plasma clozapine levels (Rostami-Hodjegan et al., 2004). Patients in both groups received concomitant psychotropic medications and medications to manage clozapine-related side effects, where clinically indicated. We extracted data on clinico-demographics, all-cause discontinuation, hospitalisation and side effects requiring physical monitoring for detection. These side effects were defined in line with guidelines (Correll et al., 2022; Taylor et al., 2021) as: hypotension = systolic blood pressure <90 mmHg; postural drop = fall in systolic blood pressure of >20 mmHg or a diastolic blood pressure of >10 mmHg within 3 min of standing; hypertension = systolic blood pressure >140 mmHg or diastolic blood pressure >90 mmHg; tachycardia = heart rate >100 per minute; and fever = temperature >37.5°C. Side effects requiring physical monitoring for detection were assessed and confirmed by clinicians during in-person reviews. We compared outcomes during the first 3 weeks of clozapine initiation, as the frequency of face-to-face monitoring do not differ between protocols after week 3.

Data were analysed using SPSS Statistics Version 28 (IBM Corp., Armonk, NY, USA). Continuous variables were tested using the Levene’s test for equality of variance, and for normality using the Shapiro-Wilk test. All data were parametric, and so the Independent Samples *t*-test was used to compare group means. Categorical variables were compared between groups using the Fisher’s exact test. All statistical tests were carried out at a two-tailed alpha-level of 0.05 with no adjustment for multiple comparisons. Clinical outcomes in the two groups were compared descriptively due to the small sample size.

Demographic and clinical data of patients who received community clozapine initiations under the LFM and SFM protocols are summarised in Table 1.

**Table 1.** Baseline clinico-demographic information.

	Low-frequency monitoring (LFM) group	S tandard-frequency monitoring (SFM) group	Comparisons statistic, *p*-value	χ^2^ values
Age^a^	41.2 ± 13.6 (16 – 61)	40.8 ± 11.8 (18 – 60)	0.940	
Sex	Male 6; Female 10	Male 6; Female 10	1.000	
Smoking status	Smoker 8; Non-smoker 8	Smoker 7; Non-smoker 9	0.723	0.125
Ethnicity	White 7; Black 7; Mixed-race 1; Asian 1	White 11; Black 4; Mixed-race 1; Asian 0	0.439	2.707
BMI^a^	30.8 ± 6.0 (22.2 – 45.3)	28.2 ± 6.8 (20.0 – 41.3)	0.268	
Type of accommodation	With family/partner 8, alone 8	With family/partner 9, alone 4, supported 2, shared 1	0.222	4.392
Initial titration/re-titration	Initial 12; re-titration 4	Initial 12; re-titration 4	1.000	
Pre-clozapine psychiatric ratings^b^				
PANSS total	79 (76 – 85)	81 (65 – 85)	0.694	
CGI	4 (3 – 5)	4 (4 – 5)	0.581	
GAF	35 (33 – 45)	40 (40 – 43)	0.890	
Side effects detected with physical monitoring^c^				
Tachycardia	3 (3)	0		
Hypotension	1 (0)	3 (0)		
Postural drop	3 (3)	5 (5)		
Hypertension^d^	2 (2)	1 (0)		
Fever	1 (0)	0		

BMI: body mass index; CGI: Clinical Global Impression scale; GAF: Global Assessment of Functioning scale; PANSS: Positive and Negative Syndrome Scale.

a Shown as mean ± standard deviation (range).

b Shown as median and interquartile range. Not all patients had psychiatric ratings recorded. For the low-frequency monitoring group, *n* = 9 for PANSS and CGI; *n* = 5 for GAF. For the SFM group, *n* = 5 for PANSS; *n* = 3 for CGI and GAF.

c Shown as cases detected during monitoring (cases persisting at the end of 3 weeks).

d Cases are limited to those with new onset hypertension during the initial 3-weeks of clozapine initiation. All cases of hypertension were mild. In the low-frequency monitoring group, one patient was refered to primary care for management of hypertension, whereas the other case resolved spontaneously.

During the first 3 weeks of monitoring, there were zero cases of discontinuation in both the LFM and SFM groups. The frequency of all-cause discontinuation did not differ numerically between groups. Moreover, there were no cases of clozapine-related side effects that required hospitalisation or fatalities in either group.

Side effects detected with physical monitoring are summarised in Table 1. One patient in the LFM group presented with a 37.7°C fever on Day 1 before starting clozapine initiation. The patient did not have associated symptoms and the fever was judged to be clinically insignificant and unrelated to clozapine. Regardless of groups, these side effects did not contribute to discontinuation of clozapine. There were no cases of syncope, infection or other serious health conditions that were detected in either group.

All patients completed the first 3 weeks of titration under the LFM protocol. This was the same proportion as in the SFM protocol, and higher than reported in previous studies of community clozapine initiation (Butler et al., 2022), providing preliminary evidence that the approach is safe for the majority of patients. The LFM protocol resulted in five fewer in-person reviews per patient during the first 3 weeks of clozapine initiation. This amounts to approximately 150 min of face-to-face contact avoided between patients and clinicians per patient, and additional inter-personal contact avoided while patients were waiting in reception. Despite this reduction, there was no evidence that LFM missed symptomatic tachycardia, hypotension, hypertension, syncope or fever, although, given the small sample size, further studies are warranted to confirm this. The relevance of the LFM protocol after the COVID-19 pandemic will depend on whether safety against SFM can be replicated in a larger cohort of patients. If this can be achieved, the protocol may provide a less burdensome, less expensive schedule for patients and staff involved in community clozapine treatment.

The main limitation of this study is the small sample size. While the study included data from all 16 patients who had undergone community initiations of clozapine under the LFM protocol at the time of the clinical audit, the findings need to be replicated in larger studies given the inter-individual variability in clozapine response. Furthermore, there is a risk of underestimating the effects of delayed detection of adverse events, particularly rare but serious side effects. Second, the LFM protocol was implemented at a single site. The feasibility of this approach should be tested in a wider range of community services while assessing additional outcomes such as subjective experiences of patients and cost-effectiveness. Third, our findings are limited to patients with moderate severity of illness, who were clinically judged to be well enough to engage with monitoring requirements in the community and lack significant risk. Finally, one of our secondary outcomes was hospitalisation due to clozapine-related side effects, which may have been biased to favour the LFM group. While patients in both groups received in-person reviews multiple times per week, and the standard of referring a patient to urgent care due to severe side effects would not have differed between observation periods, patients may have been less likely to self-refer to urgent care or agree to hospitalisation during the COVID-19 pandemic due to risk of infection.

To conclude, we have reported preliminary evidence regarding the safety of our low-frequency monitoring protocol for clozapine initiations in the community. Safety of this monitoring schedule should be systematically studied in a larger cohort of patients before being applied to a wider range of community services. Future studies should aim to compare the detection of side effects using more standardised pro forma and scales such as the Glasgow Antipsychotic Side-effects Scale for Clozapine (Hynes et al., 2015). Further work is warranted on the adherence, cost-effectiveness, and subjective experiences of patients undergoing titrations under the low-frequency monitoring protocol.

## Supplemental Material

sj-docx-1-jop-10.1177_02698811231171532 – Supplemental material for Low-frequency monitoring for community clozapine initiations: A comparative study relative to standard frequency assessments Supplemental material, sj-docx-1-jop-10.1177_02698811231171532 for Low-frequency monitoring for community clozapine initiations: A comparative study relative to standard frequency assessments by Yuya Mizuno, Devi L. Bridglal, Jack Coumbe, Hari McGrath, Ayush Adhikari, Emma Butler, Ilaria Bonoldi, David Taylor and Oliver D. Howes in Journal of Psychopharmacology
