# Supplementary material for: Low-frequency monitoring for community clozapine initiations: A comparative study relative to standard frequency assessments
Source: J Psychopharmacol. 2023 May 12;37(6):627–9. doi: 10.1177/02698811231171532 (PMC10291364; doi:10.1177/02698811231171532)
Supplement: sj-docx-1-jop-10.1177_02698811231171532 – Supplemental material for Low-frequency monitoring for community clozapine initiations: A comparative study relative to standard frequency assessments [file sj-docx-1-jop-10.1177_02698811231171532.docx]

**Supplementary Table 1. Comparison of low-frequency and standard-frequency physical monitoring protocols.** The dose increase schedules are examples. These may need to be adjusted based on tolerability and target dose (for example, female non-smokers and people with Asian ancestry generally need lower doses to achieve therapeutic levels (Correll et al., 2022)). In either titration, additional reviews may be necessary to manage side effects. The dose split between morning and evening may be varied depending on patient tolerability and response. Note the slower dose increase in the low-frequency monitoring protocol to account for less frequent face-to-face assessments. The two protocols do not differ in terms of blood monitoring, or the frequency of face-to-face assessments after Week 3.

|  | Low-frequency monitoring | Morning dose (mg) | Evening dose (mg) | Standard-frequency monitoring | Morning dose (mg) | Evening dose (mg) |
| --- | --- | --- | --- | --- | --- | --- |
| Week 1 | Mon # | / | 6.25 | Mon # | 6.25 | 6.25 |
|  | Tue | / | 12.5 | Tue # | 6.25 | 12.5 |
|  | Wed # | 6.25 | 12.5 | Wed # | 12.5 | 12.5 |
|  | Thu | 6.25 | 25 | Thu # | 12.5 | 25 |
|  | Fri # | 12.5 | 25 | Fri # | 25 | 25 |
|  | Sat | 12.5 | 25 | Sat | 25 | 25 |
|  | Sun | 12.5 | 37.5 | Sun | 25 | 50 |
| Week 2 | Mon # | 25 | 37.5 | Mon # | 25 | 50 |
|  | Tue | 25 | 50 | Tue # | 50 | 50 |
|  | Wed # | 25 | 62.5 | Wed # | 50 | 50 |
|  | Thu | 25 | 75 | Thu # | 50 | 75 |
|  | Fri # | 37.5 | 75 | Fri # | 50 | 75 |
|  | Sat | 37.5 | 75 | Sat | 75 | 75 |
|  | Sun | 37.5 | 87.5 | Sun | 75 | 75 |
| Week 3 | Mon # | 50 | 87.5 | Mon # | 75 | 100 |
|  | Tue | 50 | 100 | Tue | 75 | 100 |
|  | Wed | 50 | 125 | Wed # | 75 | 125 |
|  | Thu# | 50 | 125 | Thu | 75 | 125 |
|  | Fri | 50 | 125 | Fri # | 75 | 150 |
|  | Sat | 50 | 125 | Sat | 75 | 150 |
|  | Sun | 50 | 150 | Sun | 75 | 150 |
| Week 4 | Mon # | 50 | 150 | Mon # | 75 | 175 |
|  | Tue | 50 | 150 | Tue | 75 | 175 |
|  | Wed | 50 | 150 | Wed | 75 | 200 |
|  | Thu # | 75 | 150 | Thu # | 75 | 200 |
|  | Fri | 75 | 150 | Fri | 75 | 225 |
|  | Sat | 75 | 150 | Sat | 75 | 225 |
|  | Sun | 75 | 175 | Sun | 75 | 225 |

#= face-to-face assessments including physical observations (sitting and standing blood pressure, heart rate, SpO2, temperature, and respiratory rate), side-effect and mental state review.

**References**

Correll CU, Agid O, Crespo-Facorro B, et al. (2022) A Guideline and Checklist for Initiating and Managing Clozapine Treatment in Patients with Treatment-Resistant Schizophrenia. *CNS Drugs* 36(7): 659-679.
